# Clozapine Serum Concentrations Are Disrupted by SARS-CoV-2 Vaccinations

**DOI:** 10.1155/2023/9914879

**Published:** 2023-11-27

**Authors:** Xenia A. K. Kersting, Nicoletta Momtahen, Klaus Lieb

**Affiliations:** Department of Psychiatry and Psychotherapy, Johannes Gutenberg University Medical Center Mainz, Untere Zahlbacher Straße 8, 55131, Mainz, Germany

## Abstract

This paper reports the case of a 27-year-old man with paranoid schizophrenia who was finally stabilized on clozapine medication. After vaccination against severe acute respiratory syndrome coronavirus 2, serum levels of clozapine increased. It is well established that immune responses can trigger cytokine cascades affecting drug metabolism, which, in the case of clozapine treatment, can lead to severe intoxication.

## 1. Introduction

People with severe mental illness are at particular risk of (serious) infections with severe acute respiratory syndrome coronavirus 2 (SARS-CoV-2) [1, 2] and therefore have widely been prioritized for vaccination [3, 4]. In particular, patients with schizophrenia treated with clozapine are at increased risk of cardiac and respiratory complications [5, 6].

Clozapine is mainly metabolized by the cytochrome P450 enzyme CYP1A2 and transformed into the active metabolite norclozapine before other processing steps and renal excretion. This transformation is sensitive to various external factors, e.g., tobacco smoking, that often complicate the course of treatment. Furthermore, a variety of cytokines, such as interleukin-6, tumor necrosis factor-alpha, interferons, etc., involved in the systemic immune response can cause interactions with CYP1A2 leading to elevated levels of clozapine with a potential risk of intoxication and leukopenia [7, 8].

In line with these findings, recent studies reported that SARS-CoV-2 infections can interfere with clozapine treatment [9, 10]. However, not only the infection itself but also SARS-CoV-2 vaccination can be associated with an increase in clozapine levels [7, 11]. This observation, together with the need of adequate vaccination strategies for psychiatric patients, emphasizes the importance of therapeutic drug monitoring and potential adjustments of clozapine dosages [12].

Still, the reported evidence for this link between SARS-CoV-2 vaccination and increased serum clozapine levels is sparse, and furthermore, the temporal course of this effect remains poorly understood.

Here, we report on the case of a young man with refractory schizophrenia who was treated with clozapine and received two Pfizer-BioNTech messenger ribonucleic acid (mRNA) vaccines with a temporal distance of 5 weeks.

## 2. Case Presentation

**Figure 1:**
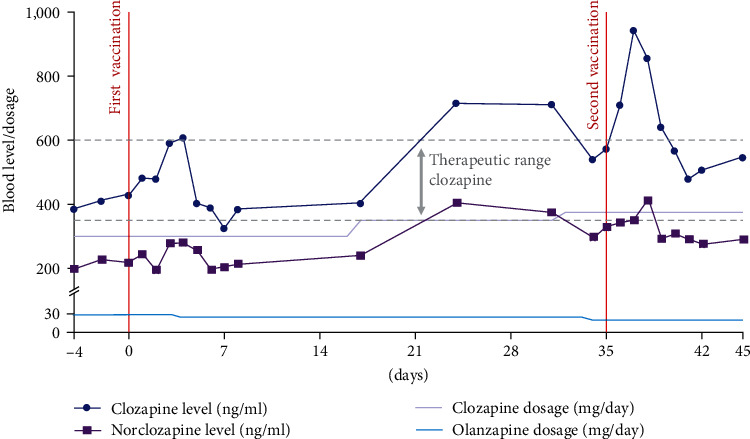
Clozapine levels, drug doses, and SARS-CoV-2 vaccinations.

A 27-year-old male patient was diagnosed with paranoid schizophrenia 1 year before the current admission to our psychiatric department. The patient was a nonsmoker and did not suffer from any concomitant illnesses. We started antipsychotic therapy with olanzapine (up to 30 mg/day), which was later combined with amisulpride (up to 1,000 mg/day) and risperidone (up to 2 mg/day), but the patient did not accomplish sufficient remission of psychotic symptoms. Therefore, we finally started a combination of olanzapine and clozapine, which led to an incipient reduction of symptoms. The serum level of clozapine reached a therapeutic steady state at a dosage of 300 mg/day (see Table 1, baseline) without sufficient clinical response. At this stage, the subject received his first dose of the Pfizer-BioNTech mRNA vaccine (COMIRNATY®) against SARS-CoV-2. We controlled the serum clozapine levels each day for the following week and observed a substantial increase, which peaked after 3–4 days and dropped back to baseline levels after 5 days. Serum norclozapine concentration followed an analogous dynamic. The patient showed only a discrete elevation of C-reactive protein (CRP), indicating a moderate immune response to the vaccination. Importantly, the patient did not develop any other adverse effects like fever, myalgia, or alteration of leukocytes. Due to serum clozapine levels that were hardly in the therapeutic range (see Figure 1), we increased the daily dosage in the first step to 350 mg/day (day 17) after having already reduced olanzapine to 25 mg/day in order to minimize additive anticholinergic side effects. As we expected clozapine levels to drop to the border-therapeutic range after normalization of cytokines, we increased the clozapine dosage yet another time to 375 mg/day (day 32) in order to prevent a loss of efficacy. In parallel, we further reduced olanzapine to 20 mg/day. Under this medication, we found clozapine levels to drift around the upper therapeutic threshold. Five weeks after the first vaccination, the subject received a second dose of the Pfizer-BioNTech mRNA vaccine, which led to a similar dynamic in serum clozapine level, however, with a larger peak after 2–3 days. The serum level stabilized again within the therapeutic range after 5 days. During the increase, we again found concomitant CRP elevation without changes in white blood cells or clinical symptoms. During the postvaccination peaks, also the ratio of serum clozapine and daily clozapine dosage showed increased values, indicating the potential of adverse side effects.

## 3. Discussion

Our reported case illustrates an association between SARS-CoV-2 vaccination and increased serum clozapine levels. Under frequent therapeutic drug monitoring in an inpatient setting, the fluctuations of clozapine levels after the first and second SARS-CoV-2 vaccinations could be observed and reacted to by adjusting the dose.

It is well established that immune responses can trigger cytokine cascades affecting drug metabolism, which, in the case of clozapine treatment, can lead to severe intoxication [5, 10, 13]. The concrete molecular pathways of these effects are not yet fully understood and remain highly unpredictable in clinical settings [7].

However, different causes of immune response vary in their potential of interacting with drug metabolism. For instance, infection with influenza virus associated with increased CRP levels can lead to altered clozapine metabolism, whereas vaccination against influenza has not shown such effects [14]. Similarly, although SARS-CoV-2 infection seems to affect clozapine metabolism, it remains unclear if vaccination has a similar effect. In our patient, we observed a moderate increase of CRP after the vaccination, concomitant to the clozapine increase. In addition, the rise of serum clozapine levels after the second vaccination showed a larger magnitude compared to the first one. We do not consider the increase in dosage to be mainly responsible for this. Typically, the immune response after the second dose of vaccination is stronger due to antibodies generated after the first vaccination.

Regular drug monitoring helps to detect and react to adverse effects at an early stage, although in our case and in a similar cohort of 139 patients, no severe granulocytopenia or agranulocytosis occurred [15]. Gee and Taylor [16] found, in addition to a reduction of neutrophils in patients with COVID-19 infection under clozapine therapy, that hardly any plasma-level controls were carried out during clozapine treatment. It is reported elsewhere that the examination intervals of special patients were extended due to hygiene considerations in the context of the pandemic [12].

## 4. Conclusion

When administering vaccination against SARS-CoV-2, it is essential to point out possible interactions due to the triggered immune response. In patients treated with clozapine, the intervals between both therapeutic drug monitoring and blood cell counts should be shortened. It would be urgently advisable to include appropriate references to the modulation of CYP1A2 and other metabolic pathways by the vaccines in the corresponding professional information for the respective medicinal product. The necessity of regular therapeutic drug monitoring and blood cell count is also evident during pandemic conditions and must be carried out in compliance with hygiene regulations. We here refer to the Consensus statement on the use of clozapine during the COVID-19 pandemic” [17]. Further research is needed to better understand the interactions between clozapine levels and vaccination against SARS-CoV-2 and to provide guidelines for dose adjustment.

## Figures and Tables

**Table 1:** Relevant results of blood analyses.

	Reference range	Baseline (d − 4)	Vacc1 (d0)	3d post-Vacc1 (d3)	6d post-Vacc1 (d6)	Recovery (d17)	Vacc2 (d35)	3d post-Vacc2 (d38)	6d post-Vacc2 (d41)
Clozapine (ng/ml)	350–600	383	426	589	387	401	571	**854**	577
Norclozapine (ng/ml)	—	197	218	277	195	239	325	411	290
Clozapine blood-dosage ratio ((ng/ml)/(mg/day))	0.6–1.2	**1.28**	**1.42**	**1.96**	**1.29**	1.15 ^*∗*^	**1.52 ^*∗*^**	**2.28**	**1.27**
Leukocytes (/nl)	3.5–10	6.93	—	4.85	—	7.2	—	4.92 ^*∗∗*^	—
Neutrophils (%)	43–75	54.5	—	58.7	—	66.9	—	52.9 ^*∗∗*^	—
CRP (mg/l)	<5	3.5	—	**12**	—	1.8	—	**14 ^*∗∗*^**	—

Bold letters indicate values above reference. d, day; Vacc1, first vaccination; Vacc 2, second vaccination.  ^*∗*^Indicates the absence of a steady state since the clozapine dosage has been changed in the previous 5 days.  ^*∗∗*^Indicates data from 4 days post Vacc2 (d39) since no routine was obtained from d38.

## Data Availability

All data analyzed in this case report are included in this published article.
